# Detection of Highly Pathogenic Avian Influenza Virus H5N1 Clade 2.3.4.4b in Great Skuas: A Species of Conservation Concern in Great Britain

**DOI:** 10.3390/v14020212

**Published:** 2022-01-21

**Authors:** Ashley C. Banyard, Fabian Z. X. Lean, Caroline Robinson, Fiona Howie, Glen Tyler, Craig Nisbet, James Seekings, Stephanie Meyer, Elliot Whittard, Henry F. Ashpitel, Mehmet Bas, Alexander M. P. Byrne, Tom Lewis, Joe James, Levon Stephan, Nicola S. Lewis, Ian H. Brown, Rowena D. E. Hansen, Scott M. Reid

**Affiliations:** 1 Animal and Plant Health Agency (APHA), Weybridge KT15 3NB, Surrey, UK; fabian.lean@apha.gov.uk (F.Z.X.L.); james.seekings@apha.gov.uk (J.S.); stephanie.meyer@apha.gov.uk (S.M.); elliot.whittard@apha.gov.uk (E.W.); henry.ashpitel@apha.gov.uk (H.F.A.); mehmet.bas@apha.gov.uk (M.B.); alexander.byrne@apha.gov.uk (A.M.P.B.); thomas.lewis@apha.gov.uk (T.L.); joe.james@apha.gov.uk (J.J.); nilewis@rvc.ac.uk (N.S.L.); ian.brown@apha.gov.uk (I.H.B.); rowena.hansen@apha.gov.uk (R.D.E.H.); scott.reid@apha.gov.uk (S.M.R.); 2 SRUC Veterinary Services, Pentlands Science Park, Bush Loan, Penicuik EH26 0PZ, Midlothian, UK; Caroline.Robinson@sruc.ac.uk (C.R.); fiona.howie@sruc.ac.uk (F.H.); 3 NatureScot, Great Glen House, Leachkin Road, Inverness IV3 8NW, UK; glen.tyler@nature.scot; 4 Fair Isle Bird Observatory, Fair Isle ZE2 9JU, Shetland, UK; 5 National Trust for Scotland, Gerinish, South Uist HS8 5RW, Western Isles, UK; cnisbet@nts.org.uk; 6 Veterinary Exotic Notifiable Disease Unit, APHA, Nobel House, London SW1P 3JR, UK; levon.stephan@apha.gov.uk; 7 Department of Pathobiology and Population Sciences, Royal Veterinary College, Hatfield AL9 7TA, Hertfordshire, UK

**Keywords:** highly pathogenic avian influenza, conserved species, H5N1, transmission, outbreak

## Abstract

The UK and Europe have seen successive outbreaks of highly pathogenic avian influenza across the 2020/21 and 2021/22 autumn/winter seasons. Understanding both the epidemiology and transmission of these viruses in different species is critical to aid mitigating measures where outbreaks cause extensive mortalities in both land- and waterfowl. Infection of different species can result in mild or asymptomatic outcomes, or acute infections that result in high morbidity and mortality levels. Definition of disease outcome in different species is of great importance to understanding the role different species play in the maintenance and transmission of these pathogens. Further, the infection of species that have conservation value is also important to recognise and characterise to understand the impact on what might be limited wild populations. Highly pathogenic avian influenza virus H5N1 clade 2.3.4.4b has been detected in great skuas (*Stercorarius skua*) across different colonies on islands off the shore of Scotland, Great Britain during summer 2021. A large number of great skuas were observed as developing severe clinical disease and dying during the epizootic and mortalities were estimated to be high where monitored. Of eight skuas submitted for post-mortem examination, seven were confirmed as being infected with this virus using a range of diagnostic assays. Here we overview the outbreak event that occurred in this species, listed as species of conservation concern in Great Britain and outline the importance of this finding with respect to virus transmission and maintenance.

## 1. Introduction

Avian influenza virus (AIV) subtype H5Nx clade 2.3.4.4 has caused extensive outbreaks across the globe during the autumn/winter 2020/2021 period [1]. Whilst the factors associated with the outcome of infection with AIV in different avian species remain undefined, the impact on wild bird species has been significant [1,2,3]. During this period, H5Nx AIVs have been associated with (through nucleic acid detection) over 300 wild bird deaths in Great Britain (GB) alone, with a diverse range of viral genotypes being involved, including H5N8, H5N5, and H5N1 [4]. In the absence of resource intensive active surveillance initiatives, the impact on wild bird populations is only possible to estimate where die-offs are observed, and this often relies on passive surveillance of citizen science observations undertaken as recreational activity or in some cases by conservation site managers or wildlife reserve wardens as an exceptional part of their usual duties. The seasonality of AIV infection in wild bird populations has meant that the peak of both AIV related mortalities and positive detections of H5Nx occurred over the winter season in the Northern hemisphere, with the last GB detection of HPAIV in a wild bird reported on 31 March 2021.

The great skua (*Stercorarius skua*), also commonly called ‘bonxies’ in GB, are apex predators that have a wide diet based predominantly on scavenging activities [5,6,7]. These include predation on other birds and stealing the prey of other species. Few other birds threaten their populations, with only larger raptors (e.g., eagles) posing a threat to breeding successes and population maintenance. Alongside feeding on fish stolen from other seabirds, these birds may also become cannibalistic where food sources are scarce, being opportunistic scavengers [5]. 

Populations of great skuas are known to be present across Norway, Iceland, the Faroe Islands, the northwest of Ireland and both mainland Scotland and the islands off the Scottish coast. Breeding sites are defined across various island regions, but population numbers and structures are infrequently assessed. As of 2004, the great skua breeding pair numbers were estimated to be approximately 8900 in Scotland, 500 on the Faroe Islands [8], 5400 in Iceland [9], 360 in Norway (including Bear Island, Svalbard and Jan Mayen [9]) and a small number in Russia (at least 10 pairs [10]). However, it is understood that the majority of the great skua breeding population resides almost entirely across selected islands off the Northern coast of GB and across other palearctic areas. This restricted breeding range includes populations across islands in the northeast Atlantic of which a high proportion inhabit the Scottish islands of Shetland and Orkney. Nesting populations aggregate on coastal moorland, with off-duty or ‘club’ groups often close to freshwater bodies. In preferred areas birds can nest in close quarters, possibly just a few tens of metres apart. Population figures across the northern islands off the coast of Scotland have been estimated at 60% of the world’s population, approximately 35,000 birds in total, with large colonies being present on Hoy, Unst, Foula, Fair Isle, and Noss [11]. In general, great skua populations have been increasing in the last 100 years with the colonisation of many islands. Whilst the great skua is listed as a species of least concern on the International Union for Conservation of Nature (IUCN) red list, they are included on the amber list of birds of conservation concern 4 within GB [12]. Certainly, great skuas are currently considered to be rare breeding birds across the areas they are known to inhabit [12]. 

An emerging situation involving observational die-offs of great skuas occurred during July 2021, with cases escalating to a point where mass mortality events were seen across different great skua breeding populations affecting several islands off the Scottish mainland. This prompted a disease investigation which subsequently detected highly pathogenic avian influenza virus (HPAIV) subtype H5N1 clade 2.3.4.4b as the cause of the mortalities. We detail here the events that occurred during this period, pathological findings associated with infection, genetic characterisation of the virus involved and the impact on the population.

## 2. Materials and Methods

Surveillance activities from different organisations involved in bird conservation include NatureScot (Shetland), Fair Isle Bird Observatory, National Trust for Scotland (St Kilda), and RSPB (Flannan Isles). Reports of sick or dead birds also came from members of the public with an interest in this species of increased conservation concern. Data was collated via both mechanisms during the reported period. 

### 2.1. Pathology Investigation

Great skua carcasses were subjected to full post-mortem examination (PME) where possible. When carcasses were not suitable for PME, swabs were taken from the oropharynx and cloaca of each bird for influenza A virological testing. Samples collected were stored at −80 °C until further virological analysis or fixed in 10% neutral buffered formalin for histological evaluation. Formalin-fixed tissues were processed and stained by haematoxylin and eosin (H&E) and immunohistochemistry (IHC) using anti-influenza A nucleoprotein antibody (Statens Serum Institute, Copenhagen, Denmark), as previously described [13]. 

### 2.2. Virological Investigation

Swabs and tissues taken from great skua carcasses were assessed for influenza A virus nucleic acid using a matrix (M) gene-specific real-time reverse-transcriptase polymerase chain reaction (rRT-PCR) assay [14] followed by subtype specific rRT-PCRs [15,16,17] to determine the haemagglutinin (HA) and neuraminidase (NA) subtypes. An H5-specific pathotype PCR was also used to rapidly confirm the presence of a multi-basic HP cleavage site as detected across previous wild bird positive samples during the autumn/winter 2020/21 season. Following the detection of notifiable AIV nucleic acid, isolation of live virus was attempted as described previously using specific pathogen-free embryonated fowls’ eggs [18,19]. Where virus isolation was successful, whole-genome sequence (WGS) data was generated using an Illumina MiSeq [20]. For comparative genetic analysis, contemporary H5 2.3.4.4b virus sequences were downloaded from the GISAID EpiFlu database (https://platform.gisaid.org/ accessed on 9 August 2021). WGS were deposited on the GISAID database under accession numbers: EPI_ISL_5530613 (A/great_skua/Scotland/041672/2021), EPI_ISL_5804789 (A/great_skua/Scotland/B07779/2021) and EPI_ISL_6029360 (A/great_skua/Scotland/042505/2021).

## 3. Results and Discussion

**Figure 1:**
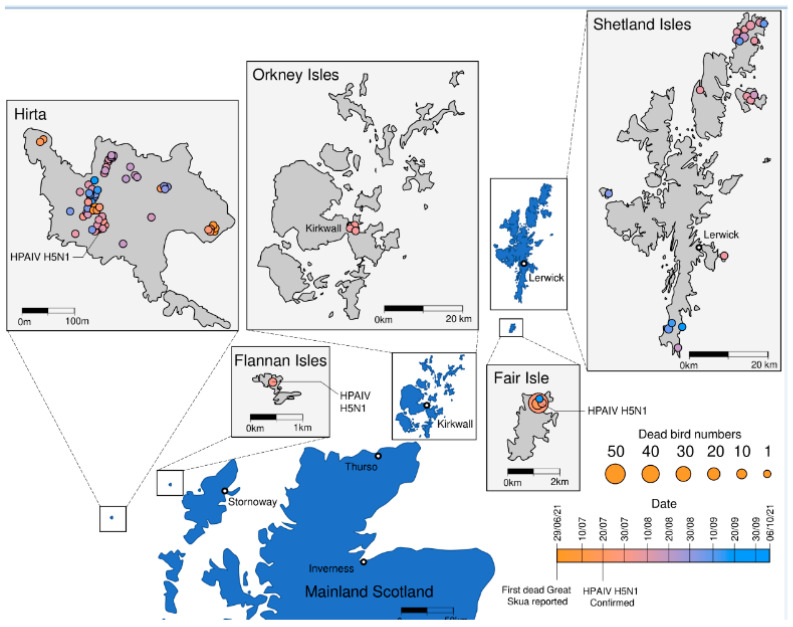
Geographical distribution of great skua carcasses and timeline of detection. The distribution of observed deaths is shown on the map for each island. Cases are coloured according to time of observation as denoted in the key. The size of circle is proportional to the number of cases with larger circles denoting a greater number of observed mortalities as per the key. Positive cases are also noted or each island.

Between the 29 June and the 2 October 2021, a number of great skua carcasses were detected through surveillance activities covering several islands off the North coast of Scotland. Skuas are monitored through various mechanisms including bird watching groups and members of the public. A timeline of observed mortalities demonstrated how dead birds were found at geographically distant sites, likely reflecting independent incursions of disease as infected individuals would not be expected to move between colonies during the months of April to July when breeding occurs (Figure 1). Clinical disease observed in skuas included head tilt, inability to fly, incoordination, and twitching of head and wings suggesting neurological impairment. Later birds were moribund and grounded. Death occurred within 24 h of being in a moribund state following local observations (Video S1).

To investigate the cause of the mortalities, swab samples, and where available, tissues from carcasses, were submitted to the Animal and Plant Health Agency (APHA), Weybridge for AIV molecular testing and pathological assessment. Frontline testing for AIV detected viral nucleic acid (M gene) positives from three of four skuas swabbed from Fair Isle (Shetland) on 20 July 2021 (Table 1). These positive swabs were then tested using subtyping PCRs for HA and NA subtype. To rapidly determine the viral pathotype, an H5 HPAIV clade 2.3.4.4 specific rRT-PCR assay was undertaken [21]. Cleavage site sequence was confirmed by whole genome sequencing [20]. Positive samples were inoculated into eggs to attempt virus isolation and an isolate was obtained from an oropharyngeal swab taken from one of the four Fair Isle great skuas (AV-21-040939). Of the four birds tested, three were positive for M-gene and H5-specific rRT-PCRs with one bird also testing positive by N1-specific rRT-PCR. All three birds were positive by H5 pathotyping rRT-PCR, thereby denoting the presence of HPAIV. Tissue samples from the three positive birds from Fair Isle were positive including liver (Bird 1—M, H5 and N1), spleen (Bird 1—H5 and H5 pathotyping), liver (bird 2—M, H5, H5 pathotyping and N1) and liver (bird 4—M, H5, H5 pathotyping and N1) (Table 1). Swabs and liver collected from bird 3 were rRT-PCR negative. During this period, clinical disease in birds, or bird deaths were observed elsewhere across the different colonies (Video S1). Further samples tested positive from birds sampled on the Flannan Isles two days later (22 July 2021) (Table 1, Figure 1) with a single great skua testing positive for M gene, H5 and N1 targets in both oropharyngeal and cloacal swabs as well as bulk viscera submitted following PME. The final detection of AIV in the skua population across the defined colonies was in birds submitted from St Kilda in the Outer Hebrides. A bird carcass collected on 29 July 2021 was positive for M gene, H5 and N1 in both oropharyngeal and cloacal swabs, and in bulk viscera. Four further birds from St Kilda that had been collected 10 days earlier were investigated retrospectively and three out of four were positive on carcass swabs for M gene, H5 or N1. Pooled standard tissues namely: brain, lung and trachea, intestinal contents and viscera from these four birds were also positive. Live virus was also isolated from the brain tissue pool from these birds (Table 1). Bulk viscera from a great black-backed gull that was found dead on the Flannan Isles was also tested alongside swabs. Swab material was negative for this bird whilst bulk viscera was positive for H5N1 HPAIV. It is likely that this finding represents ingestion of infected material and absence of other positive material precludes further definition of cause of death for this bird.

**Figure 2:**
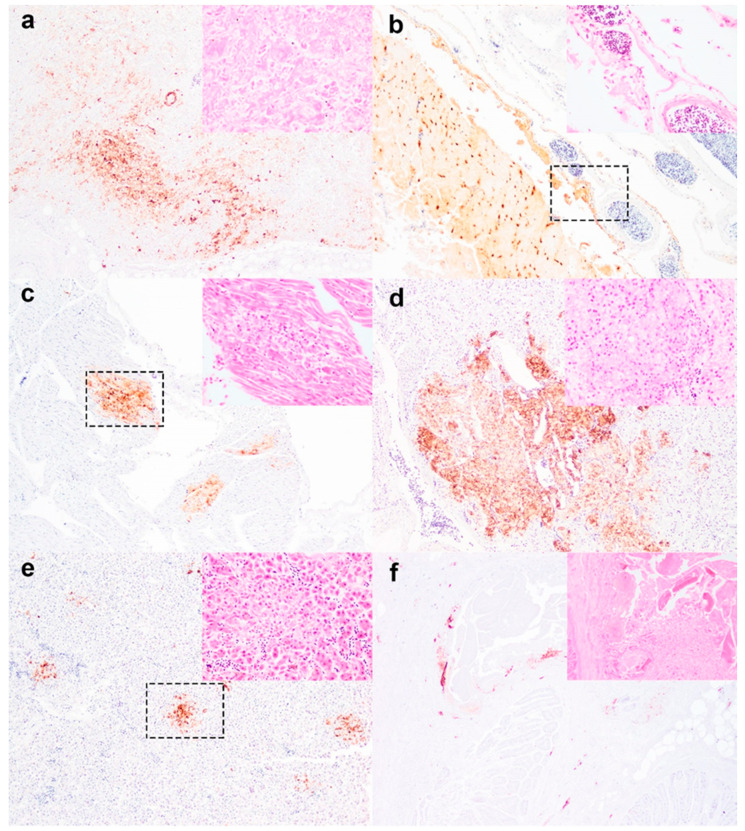
Microscopic findings of great skua infected with HPAIV H5N8. (**a**) Severe pancreatic necrosis with abundant virus antigens; (**b**) Mild non-suppurative meningoencephalitis with confluent viral immunopositive immunolabelling; (**c**) Rare myocardial necrosis associated with scattered virus antigens; (**d**) Moderate adrenal necrosis with confluent virus antigen labelling; (**e**) Rare multifocal hepatic necrosis with scattered viral immunolabelling; (**f**) A focus of glandular necrosis of the proventriculus and scattered immunolabelling within the submucosa. Region of interest within IHC images outlined by dotted line box corresponds to H&E insets. Immunohistochemical images were taken at 100× magnification. Histological images were taken at 400× magnification for a to e but 200× for the proventriculus.

A total of nine great skua carcasses were examined by APHA. Birds from Fair Isle were examined by SRUC. This comprised of five males and four females. Generally, the birds were in good body condition with occasional soiling of the vent or empty proventriculus and gizzard (n = 2) at PME. Notable microscopic changes that could be attributed to the morbidity and mortality of the skuas included severe pancreatic necrosis (n = 4) (Figure 2a), mild to moderate meningoencephalitis (n = 3) (Figure 2b), rare myocardial necrosis (n = 1) (Figure 2c), moderate adrenal necrosis (n = 1) (Figure 2d), mild hepatic necrosis (n = 1) (Figure 2e) and rare proventricular necrosis (n = 1) (Figure 2f). Incidentally, *Syngamus* sp. (gape worm) was noted in the trachea of a bird but was not associated with significant lesion at gross or microscopical level. Viral immunohistochemical analysis revealed that the viral antigens were present more widely in contrast to the distribution and degree of microscopic lesions. Virus antigens were commonly found in the heart, brain, kidney, spleen, adrenal, skeletal muscle, lung, air sac, kidney and pancreas (Table 2). Some of these immunolabelled tissues and areas can also be co-localised with areas of histopathologic changes as described above (Figure 2). Infrequently, virus antigens were also observed in the calamus, syrinx, gizzard, and duodenum (Table 2). The cellular tropism of viral infection included neuronal, vascular and epitheliotropic. Overall, the histopathologic changes associated with HPAIV were acute to peracute and the cause of death is due to multisystemic infection including neuronal (CNS tropism), cardiovascular (heart), and metabolic (pancreas, adrenal, liver).

Genetic assessment of the virus infecting the skuas established the relationship of the H5N1 HPAI virus involved with the sequences of previous H5N1 viruses detected across the UK and Europe during the 2020/21 autumn/winter AIV season (A/great_skua/Scotland/042505/2021; A/great_skua/Scotland/B07779/2021; A/great_skua/Scotland/041672/2021) All genes clustered closely with isolates detected during the 2020/21 season with 99% nucleotide similarity with the H5N1 virus detected in pheasants (*Phasianus colchicus*) in Scotland (Accession number: EPI1848936), poultry (EPI1838670), Eurasian teal (*Anas crecca*) (EPI1812373), greylag goose (*Anser anser*) (EPI1812373) and Eurasian wigeon (*Mareca penelope*) (EPI1815140) from the Netherlands. One exception was the identity of the MP segment that had closet nucleotide identity to virus isolated from red knots (*Calidris canutus*) in Germany (H5N3-EPI1850153 and EPI1850145) and the Republic of Ireland (H5N3-EPI1860026) as well as swans in Lithuania (H5N8-EPI1858593) and a greylag goose in Spain (H5N8-EPI1860066) (Supplementary Figure S1). The cleavage site (CS) sequence motif for the skua isolates was PLREKRRKRGLF as reported across the vast majority of HPAIV CS sequences determined over the 2020/21 autumn/winter season across GB and continental Europe. 

Apart from the detection of dead or diseased skuas, no further mass mortalities in island-resident bird species were observed during the reported outbreak period. One exception was the submission of a single juvenile great black-backed gull (*Larus marinus*) that tested negative for AIV nucleic acid in both oropharyngeal and cloacal swabs. However, HPAIV H5N1 was detected by rRT-PCR in bulk viscera tissue submitted from the same bird for analysis. All tissues from this bird were negative by IHC (data not shown) although brain and pancreas were not included in the submission. The predatory role of great black backed gulls may indicate that this particular bird had consumed a contaminated carcass, likely following predation on dead or diseased skuas. Of note, surveyors working in hills in central Shetland reported greylag geese dead around lochs during early August although carcasses were unavailable for testing. Furthermore, ten greylag geese, a potential prey species or birds potentially scavenged by great skuas, tested AIV-positive under the UK passive wild bird surveillance scheme during the 2020/21 season. However, from those detected across GB, none tested positive for the H5N1 genotype (8 were H5N8 and 2 were H5Nx). Only rooks (n = 3) positive for H5N1 had previously been detected in Scotland with several other species (mute swans (n = 9); Canada geese (n = 1)) including apex predators (red kite (n = 1) and buzzards (n = 1)) testing positive for H5N1 in England over the same autumn/winter 2020/21 time period. Apex predators are often observed during the tail end of outbreaks as these birds scavenge on birds that may have succumbed to HPAI. Skuas fall under the definition of apex predators owing to their piratical behaviour pattern. Certainly, throughout the event, scavenged dead skuas were detected and it is likely that once the first bird succumbed to infection within a colony, birds scavenging on the carcass facilitated spread throughout colonies. Movement of birds between islands is well documented and likely accounts for the dispersal between colonies.

## 4. Conclusions

In conclusion, the detection of sequential mass mortality events across the series of islands that harbour skua colonies demonstrates the potential threat of infectious diseases to vulnerable populations. There remains uncertainty as to whether the mortalities across multiple locations resulted following independent incursions or skua movements leading to onward introductions. However, the likelihood of skua travel between colonies during the breeding season is considered low, suggesting a potential role of independent incursions across the different colonies. The number of breeding adults on St. Kilda is approximately 500 and 1000 on Fair Isle. At both sites more that 10% of these were found dead during the outbreak, with the very likely possibility that more birds died but were not found. Breeding productivity at the main great skua colonies was noted as being very low during this period, possibly as a consequence of the HPAI outbreak. Alongside observed mortalities, the impact of the outbreak on populations, through the death of a parent bird and subsequent increased pressure to raise young, may impact future generations. Future census activities will help to define where losses have impacted across skua density on each of the islands. Gaining a better understanding of outbreaks in different species is critical to understanding the transmission dynamics of these viruses and the potential for further geographical spread. 

## Figures and Tables

**Table 1:** Overview of molecular testing of samples submitted across the disease event.

Sample Location (and Collection Date)	Species	Sample ID or Bird Number	Sample Type	Real-Time RT-PCR				Interpretation (and GISAID Reference)
				M Gene ^a^	H5 HA2 ^b^	H5 HP ^c^	N1 ^d^	
Fair Isle, Shetland 20 July 2021	Great Skua (Bird 1)	AV-21-040939	OP swab	+ve	+ve	+ve	+ve	HPAIV H5N1 (EPI_ISL_5530613)
		97670002	Liver	+ve	+ve	+ve	+ve	HPAIV H5N1
		97670002	Spleen	−ve	+ve	+ve	−ve	HPAIV H5Nx
	Great Skua (Bird 2)	AV-21-040942	C swab	+ve	+ve	+ve	−ve	HPAIV H5Nx
		97670003	Liver	+ve	+ve	+ve	+ve	HPAIV H5N1
	Great Skua (Bird 4)	AV-21-040945	OP swab	+ve	+ve	+ve	−ve	HPAIV H5Nx
		97670005	Liver	+ve	+ve	+ve	+ve	HPAIV H5N1
Flannan Isles, Outer Hebrides 22 July 2021	Great Skua	AV-21-042505	OP swab	+ve	+ve	+ve	+ve	HPAIV H5N1 (EPI_ISL_6029360)
		AV-21-042506	C swab	+ve	+ve	+ve	+ve	HPAIV H5N1
		50625976	Bulk viscera	+ve	+ve	+ve	+ve	HPAIV H5N1
	Black-Backed Gull	AV-21-042507	OP swab	−ve	−ve	−ve	−ve	AIV-negative
		AV-21-042508	C swab	−ve	−ve	−ve	−ve	AIV-negative
		50625977	Bulk viscera	−ve	+ve	+ve	+ve	HPAIV H5N1
St. Kilda, Outer Hebrides 29 July 2021	Great Skua	50627155	OP	+ve	+ve	+ve	+ve	HPAIV H5N1
		50627155	C	+ve	+ve	+ve	+ve	HPAIV H5N1
		50627155	Bulk viscera	+ve	+ve	+ve	+ve	HPAIV H5N1 (EPI_ISL_5804789)
St. Kilda, Outer Hebrides	Great Skua (Received and tested on 19 August 2021)	Bird 1	OP carcass swab	+ve	+ve	+ve	+ve	HPAIV H5N1
			C carcass swab	−ve	−ve	−ve	−ve	
	Carcass swab real-time RT-PCR performed on 20 August 2021	Bird 2	OP carcass swab	+ve	+ve	+ve	+ve	HPAIV H5N1
			C carcass swab	+ve	+ve	+ve	−ve	
		Bird 3	OP carcass swab	−ve	+ve	+ve	−ve	HPAIV H5Nx
			C carcass swab	−ve	−ve	−ve	−ve	
		Bird 4	OP carcass swab	−ve	−ve	+ve	−ve	HPAIV H5Nx
			C carcass swab	−ve	−ve	−ve	−ve	
	Pooled tissue (Test performed on 24 August 2021)	Birds 1–4	Brain	+ve	+ve	+ve	+ve	HPAIV H5Nx-positive pooled carcasses
			Lung and trachea	+ve	+ve	+ve	+ve	
			Intestinal contents	+ve	+ve	+ve	+ve	
			Pooled viscera	+ve	+ve	+ve	+ve	

RT-PCR assays: ^a^ [14], ^b^ [15], ^c^ [21], ^d^ [15], e = OP = oropharyngeal; C = cloacal; +ve = positive; −ve = negative.

**Table 2:** Summary of histopathology and viral immunohistochemistry findings.

Tissue	Number Examined	Histopathology ^a^		Viral Immunohistochemistry ^b^	
		Percent (Number of Cases)	Grade	Percent (Number of Cases)	Grade
**Heart**	9	11(1)	+	67 (6)	+ to +++
**Skeletal muscle**	6	0 (0)	−	50 (3)	+, ++
**Skin**	5	0 (0)	−	0 (0)	−
**Feather**	5	0 (0)	−	20 (1)	++
**Brain**	6	50 (3)	+, ++	83 (5)	++ to ++++
**Kidney**	7	0 (0)	−	71 (5)	+, ++
**Spleen**	3	0 (0)	−	100 (3)	+, ++, ++++
**Adrenal**	2	100 (2)	++, +++	100 (2)	++, +++
**Trachea**	8	0 (0)	−	0 (0)	−
**Syrinx**	4	0 (0)	−	25 (1)	+
**Lung**	9	0 (0)	−	78 (7)	+, ++, ++++
**Air sac**	6	0 (0)	−	50 (3)	+, ++
**Proventriculus**	8	13 (1)	+	38 (3)	+, ++
**Gizzard**	6	0 (0)	+	17 (1)	+, ++
**Liver**	8	13 (1)	++	63 (5)	+, ++
**Pancreas**	4	100 (4)	++++	100 (4)	++ to ++++
**Duodenum**	7	0 (0)	−	29 (2)	+, ++
**Colon**	5	0 (0)	−	0 (0)	−

^a^ Histopathology grade: Absent −, minimal +, mild ++, moderate +++, severe ++++; ^b^ Immunohistochemical grade: Absent −, rare +, scattered ++, confluent +++, abundant ++++.

## Supplementary Materials

The following supporting information can be downloaded at: https://www.mdpi.com/article/10.3390/v14020212/s1, Figure S1: Maximum likelihood phylogenetics trees of the HA, NA, PB2, PB1, PA, NP, NA, MP and NS genes; Video S1: Clinical disease observed in the great skua.
