# Supplementary material for: Detection of Highly Pathogenic Avian Influenza Virus H5N1 Clade 2.3.4.4b in Great Skuas: A Species of Conservation Concern in Great Britain
Source: Viruses. 2022 Jan 21;14(2):212. doi: 10.3390/v14020212 (PMC8878110; doi:10.3390/v14020212)
Supplement: Supplementary file 1 [file viruses-14-00212-s001.zip › viruses-1570205-supplementary.pdf]

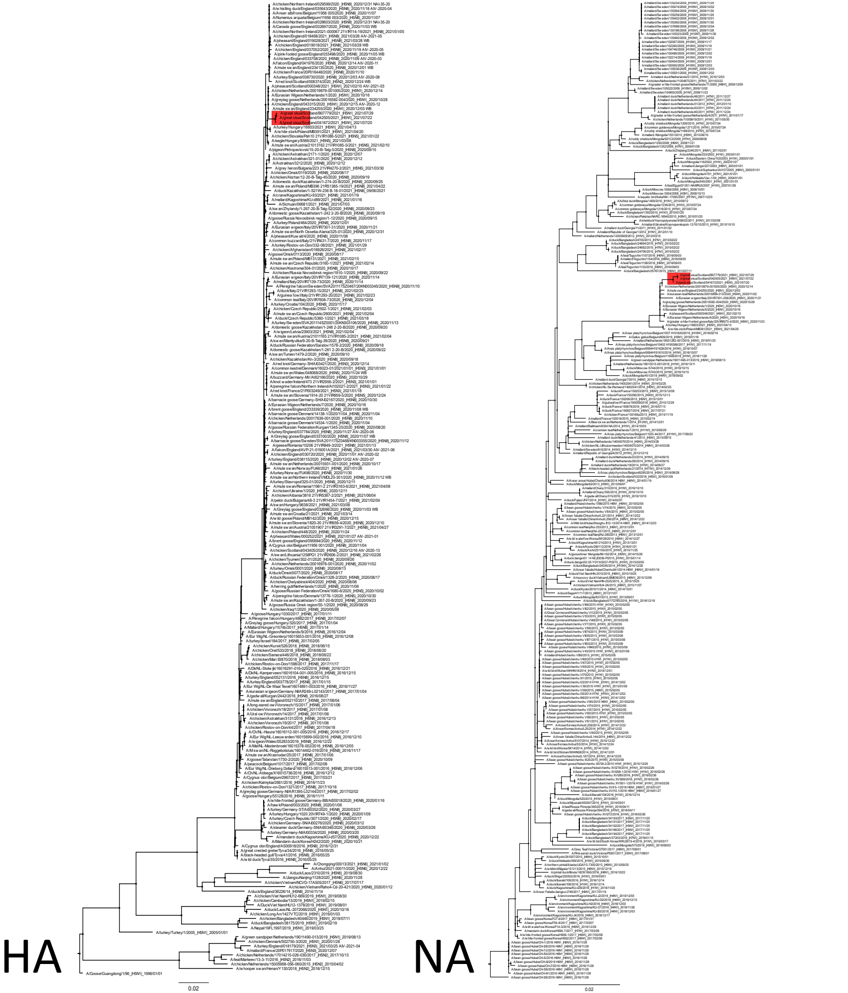

HA
0.02
NA
0.02

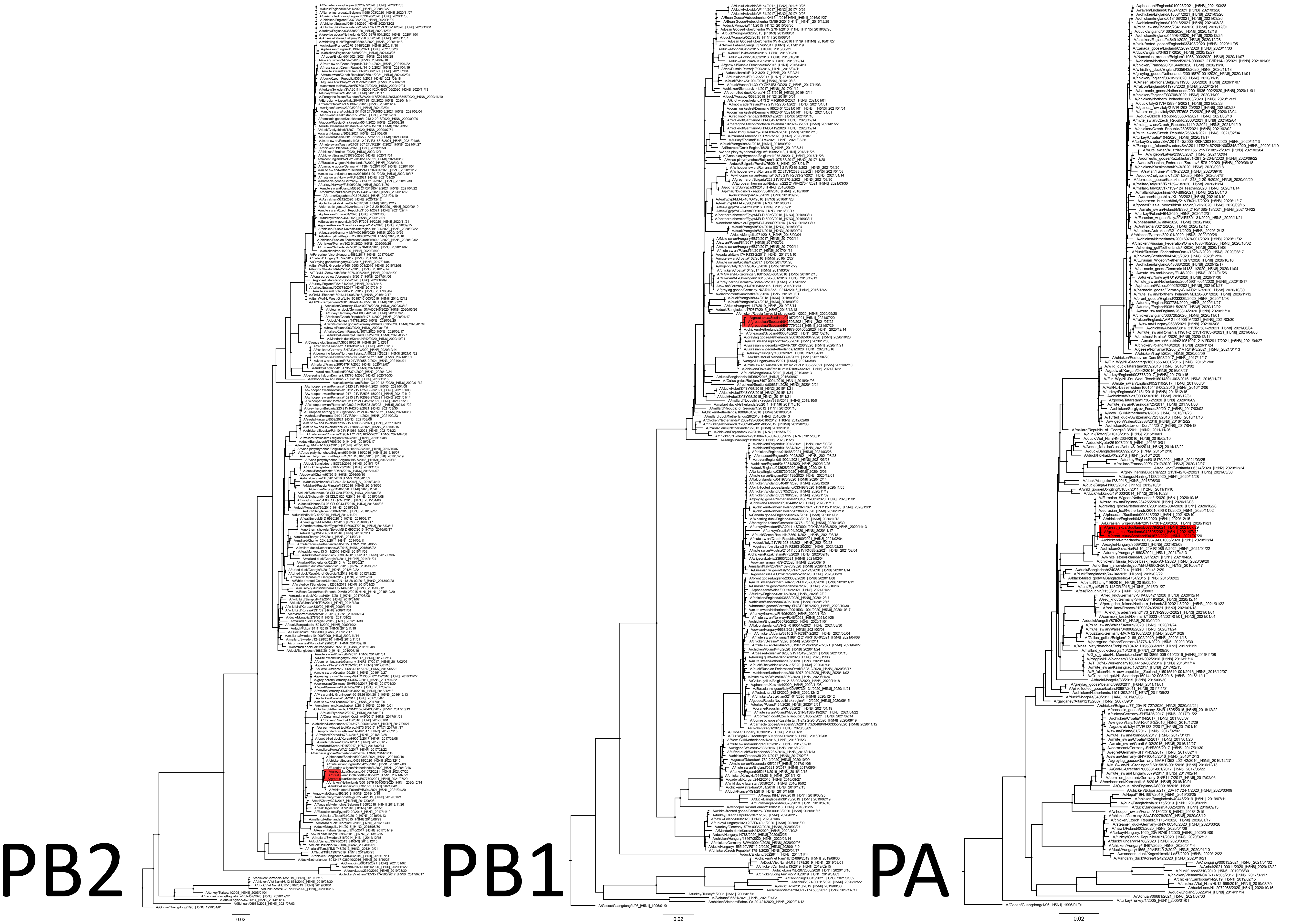

PB2
PB1
PA
0.02
0.02
0.02

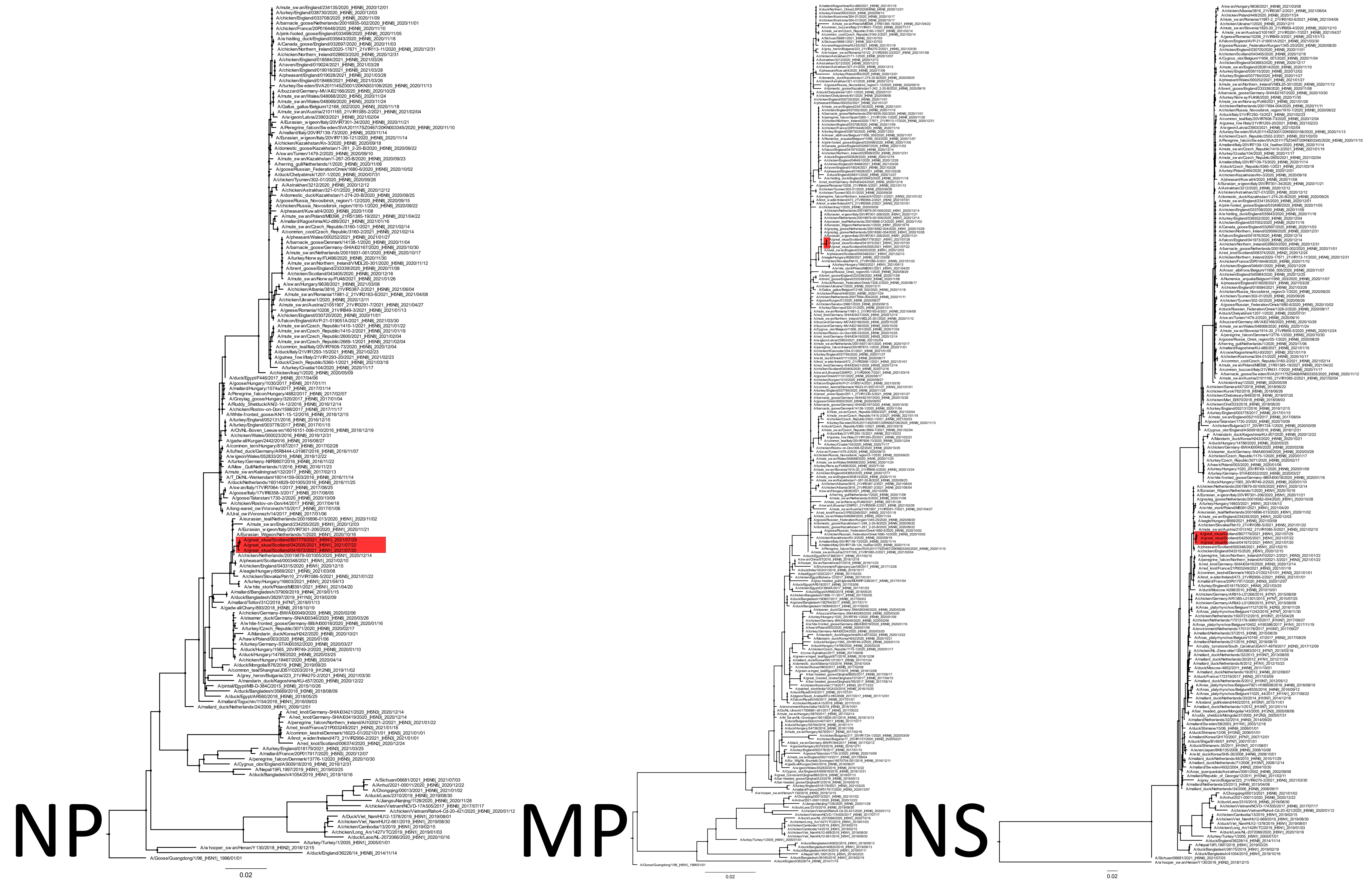
NP
0.02
MP
0.02
NS
0.02

Figure S1: Maximum likelihood phylogenetics trees of the HA, NA, PB2, PB1, PA, NP, NA, MP and NS genes. H5N1 viruses detected in Scotland in July 2021 are highlighted in red. Relationships among the Eurasian 2020/21 H5 HPAIV strains were inferred by adding the novel sequence data to that available on GISAID Epiflu on 29th July 2021. Sequences were aligned using MAFFT v7.407 and phylogenetic trees inferred using IQTree with ultrafast bootstrap node support. Trees were visualised in FigTree v1.4.4.
